# Supplementary material for: Mitral Valve Systolic Anterior Motion in Robotic Thoracic Surgery as the Cause of Unexplained Hemodynamic Shock: From a Case Report to Recommendations
Source: J Clin Med. 2022 Oct 13;11(20):6044. doi: 10.3390/jcm11206044 (PMC9604796; doi:10.3390/jcm11206044)
Supplement: Supplementary file 1 [file jcm-11-06044-s001.zip › Supplementary Materials.pdf]

Supplemental Material to

Mitral valve systolic anterior motion in robotic thoracic surgery as the cause of unexplained hemodynamic shock: from a case report to recommendations.

Fabrizio Monaco, Filippo D'Amico, Gaia Barucco, Margherita Licheri , Pierluigi Novellis, Paola Ciriaco, Veronesi Giulia

Supplementary Video S1. Transesophageal long axis view. Systolic anterior motion of the anterior leaflet in the left ventricle outflow track and premature closure of the aortic valve during the systole.

Supplementary Video S2. Transesophageal long axis view with color Doppler showing aliasing in the left ventricle outflow track and severe mitral regurgitation due to systolic anterior motion of the mitral anterior leaflet.

Supplementary Video S3. Transesophageal deep transgastric view with color Doppler showing mitral regurgitation and left ventricle outflow tract obstruction at the same time. This is the best view to assess the degree of left ventricle outflow tract obstruction.

Supplementary Figure S1. Deep transgastric view and continuous Doppler to assess the degree of left ventricle outflow tract obstruction (instantaneous gradient 179 mmHg).
