# Supplementary figures and images for: Mitral Valve Systolic Anterior Motion in Robotic Thoracic Surgery as the Cause of Unexplained Hemodynamic Shock: From a Case Report to Recommendations
Source: J Clin Med. 2022 Oct 13;11(20):6044. doi: 10.3390/jcm11206044 (PMC9604796; doi:10.3390/jcm11206044)

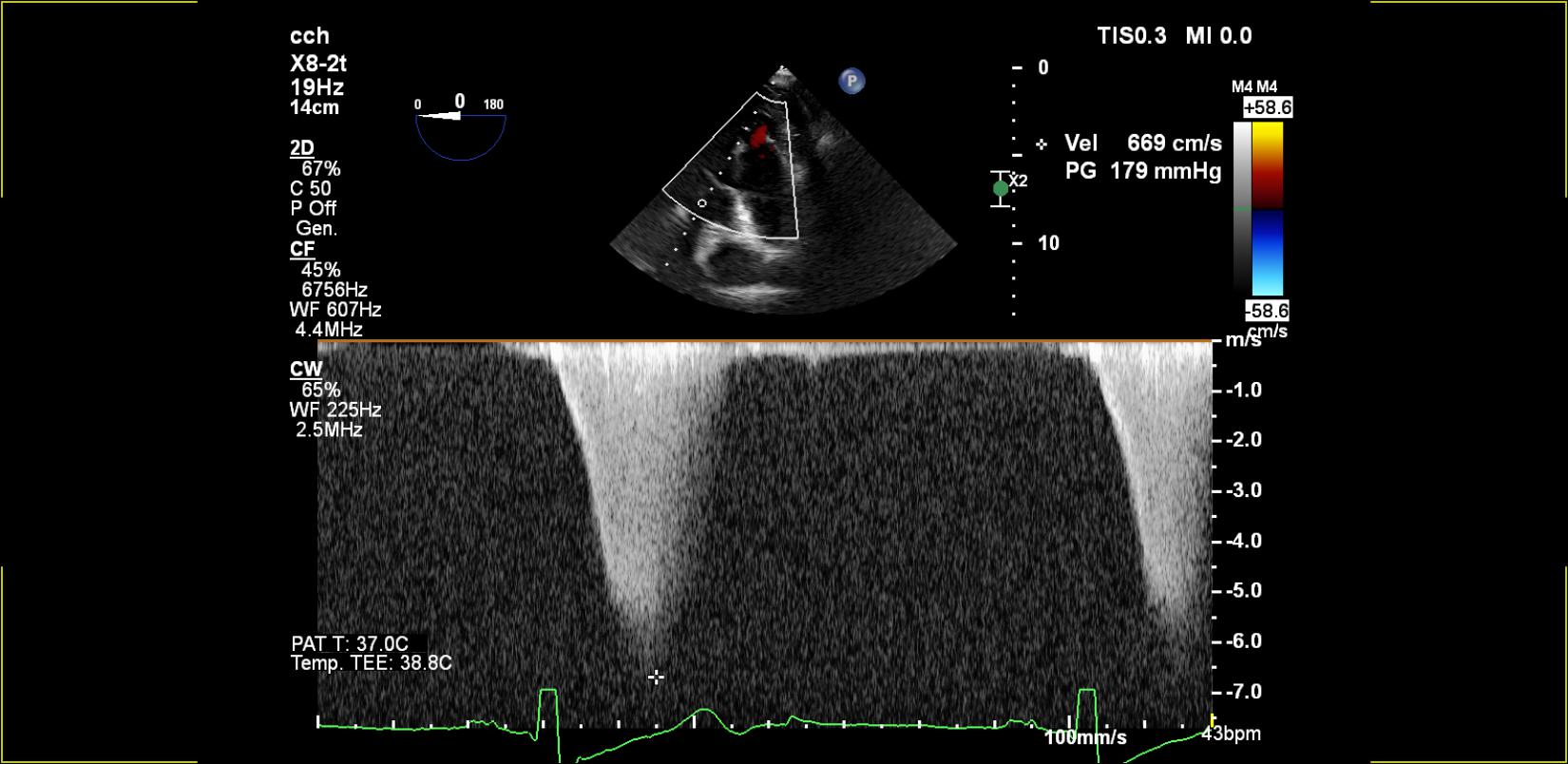

Supplement: Supplementary file 1 [file jcm-11-06044-s001.zip › Supplementary Figure S1.jpg]
